# A short review of recent advances in CO_2_ hydrogenation to hydrocarbons over heterogeneous catalysts

**DOI:** 10.1039/c7ra13546g

**Published:** 2018-02-16

**Authors:** Wenhui Li, Haozhi Wang, Xiao Jiang, Jie Zhu, Zhongmin Liu, Xinwen Guo, Chunshan Song

**Affiliations:** State Key Laboratory of Fine Chemicals, PSU-DUT Joint Center for Energy Research, School of Chemical Engineering, Dalian University of Technology Dalian 116024 P. R. China; National Engineering Laboratory for Methanol to Olefins, Dalian National Laboratory for Clean Energy, Dalian Institute of Chemical Physics, Chinese Academy of Sciences Dalian 116023 P. R. China; Clean Fuels & Catalysis Program, EMS Energy Institute, PSU-DUT Joint Center for Energy Research, Departments of Energy and Mineral Engineering and Chemical Engineering, Pennsylvania State University University Park PA 16802 USA guoxw@dlut.edu.cn csong@psu.edu

## Abstract

CO_2_ hydrogenation to hydrocarbons is a promising way of making waste to wealth and energy storage, which also solves the environmental and energy issues caused by CO_2_ emissions. Much efforts and research are aimed at the conversion of CO_2_*via* hydrogenation to various value-added hydrocarbons, such as CH_4_, lower olefins, gasoline, or long-chain hydrocarbons catalyzed by different catalysts with various mechanisms. This review provides an overview of advances in CO_2_ hydrogenation to hydrocarbons that have been achieved recently in terms of catalyst design, catalytic performance and reaction mechanism from both experiments and density functional theory calculations. In addition, the factors influencing the performance of catalysts and the first C–C coupling mechanism through different routes are also revealed. The fundamental factor for product selectivity is the surface H/C ratio adjusted by active metals, supports and promoters. Furthermore, the technical and application challenges of CO_2_ conversion into useful fuels/chemicals are also summarized. To meet these challenges, future research directions are proposed in this review.

## Abstract 2

CO_2_ hydrogenation to hydrocarbons over heterogeneous catalysts.

## Graphical abstract

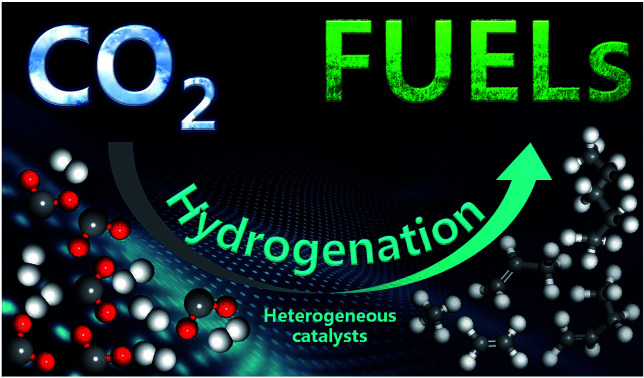

## 1. Introduction

Continuing consumption of fossil fuels worldwide has led to an increasing CO_2_ concentration in the atmosphere, and global climate change caused by greenhouse gases has become a major challenge. Mitigation of CO_2_ concentration in the atmosphere is in urgent need due to the continuing rise in atmospheric CO_2_ concentration (*e.g.*, exceeding 400 ppm in 2016 (ref. 1)) and its negative and even possibly irreversible impact on the climate system. A recent report by UNEP (United Nations Environment Programme) estimated that if no firm global action is taken against climate change, temperatures might increase by more than 2 °C by 2050, and more than 4 °C by 2100.^2^ In order to avoid this outcome, scientists indicate that global greenhouse gas emissions need to be reduced by at least 50% by 2050 compared to 1990, while the European Commission objective aims to achieve a reduction of 80–95% greenhouse gas emissions by 2050 compared to 1990.^2^ A number of Europe's key partners from all over the world, such as China, Brazil, and Korea, are addressing these issues through concrete actions to promote the “low carbon economy”.^2^ The oil company TOTAL has generated its climate strategy based on the International Energy Agency's 2 °C scenario which aims to limit emissions to approximately 15 Gt CO_2_-eq. per year in 2050 with the objective to achieve carbon neutrality in the second half of the century.

At present, CO_2_ can be reduced in three ways: control of CO_2_ emissions, CO_2_ capture and storage, and chemical conversion and utilization of CO_2_.^3,4^ Carbon storage is important for cutting CO_2_ emissions quickly, but has an issue of potential leakage of CO_2_.^4,5^ CO_2_ can be regarded as a carbon source to offer an alternative to produce carbon-containing value-added products and feedstocks. CO_2_ obtained by capture not only can provide a pure carbon source for hydrogenation, but also can avoid the leakage problem caused by CO_2_ storage. In addition, the National Aeronautics and Space Administration (NASA) also regarded the Sabatier reaction (CO_2_ methanation) as a step in reclaiming oxygen within closed cycle life support systems.^6,7^ Even the CO_2_ in industrial flue gas can be used directly as a feed for hydrogenation.^8^ Therefore, an efficient utilization of renewable carbon resources is crucial and beneficial to maintain a long-term and sustainable development of our society. CO_2_ conversion requires energy input, and its conjunction with renewable energy would make this strategy more promising in terms of sustainability and environmental friendliness.

CO_2_ reduction can be catalyzed through electrocatalysis,^9^ photocatalysis,^10^ and thermal catalysis. Among them, thermal catalysis receives significant attention due to its fast kinetics and flexible combination of active components. Carbon dioxide is a highly stable molecule, the activation and subsequent conversion of which alone are energy demanding. The addition of another substance with relatively higher Gibbs energy will make the CO_2_ conversion more favorable thermodynamically. However, electrocatalysis and photocatalysis have the fatal flaw of low energy efficiency. Therefore, CO_2_ hydrogenation^11–14^ using H_2_ produced with renewable energy sources^15,16^ is a promising research direction to produce chemicals and fuels,^17–24^ which not only reduces the CO_2_ emissions, but also covers the shortage of fossil fuels.

**Fig. 1:**
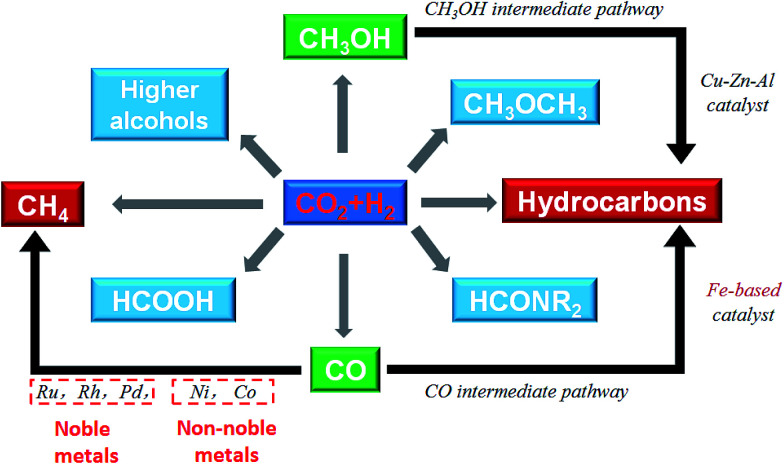
Conversion of CO_2_ to chemicals and fuels through hydrogenation.

Catalytic hydrogenation of CO_2_ using H_2_ produced with renewable energy is considered as a potential path forward for the sustainable production of lower olefins,^25^ higher hydrocarbons,^26^ formic acid,^27^ methanol,^28,29^ and higher alcohols^30^ (Fig. 1). Considering the depletion of fossil fuels, CO_2_ hydrogenation to hydrocarbons is a promising way to covert CO_2_ into fuels among the other CO_2_ hydrogenation paths. Yet, we need to confront two challenges along with it: (1) sustainable hydrogen source and (2) dispersed product distribution. Much effort has been devoted to solving the former challenge, and scientists have already made great progress in water electrolysis to produce H_2_ using electricity generated with solar or wind or other renewable energy, and water splitting using photocatalytic, photoelectrochemical or other photochemical processes. There are established industrial technologies for water electrolysis with energy efficiencies of around 70%.^31^ However, the C_2+_ hydrocarbons have a wide distribution. For example, CH_4_, C_2_–C_4_, and C_5+_ are targeted regions for production, while the selectivity was spread in a wide range, which becomes an obstruction to meet the requirement for real applications in industry. However, to date few reviews have dealt with the CO_2_ conversion mechanism and hydrocarbon chain growth both experimentally and with density functional theory (DFT) calculations. This review provides an overview of advances in CO_2_ hydrogenation to hydrocarbons that have been achieved recently in terms of catalyst design, catalytic performance and reaction mechanism from both experiments and DFT calculations. The review is organized based on some apparent factors which affect catalyst performance and unified to the essential reason for CO_2_ hydrogenation that is the chemical state of the catalysts. The fundamental factor for product selectivity is the surface H/C ratio adjusted by the use catalysts. In addition, DFT research advances are summarized from the point of view of C–O bond cleavage and C–C bond formation which gave a deep insight into CO_2_ activation and conversion. Guidance as to how to adjust the catalysts to promote hydrocarbon chain growth in CO_2_ conversion is also given in this review.

## 2. CO_2_ hydrogenation to CH_4_

**Fig. 2:**
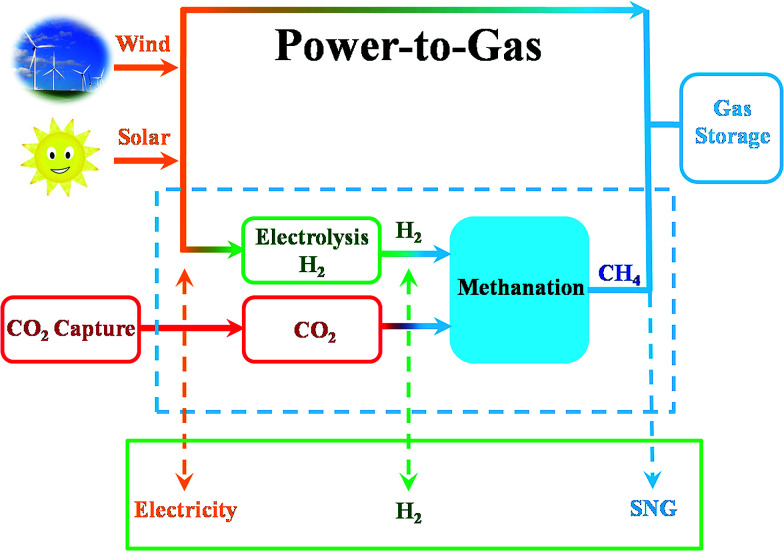
Schematic illustration of CO_2_-based sustainable production of chemicals and fuels.

CO_2_ also can be identified as an energy carrier for the transformation of renewable energy. As aforementioned, CO_2_ hydrogenation to value-added products is one of the promising approaches to combat the CO_2_-induced climate change, wherein the electrolysis of water to generate H_2_ with renewable energy is a potential energy storage approach, and would definitely add more credits to the establishment of such a sustainable carbon-based cycle. However, currently uses of renewable energy sources are limited by their inherent intermittency, and require scalable means of storage.^32^ Therefore, the production of synthetic natural gas or liquid fuels is the most feasible and convenient way to store large amounts of intermittent energy produced from renewable sources for long periods. Among them, the so-called ‘‘power to gas’’ (PtG) concept has garnered significant attention (Fig. 2),^33^ in which CO_2_ reacts with H_2_, generated by water electrolysis with renewable wind or solar energy, to produce CH_4_ as an alternative source of natural gas. In Copenhagen, a commercial scale operation PtG project with 1.0 MW capacity was running successfully using transformation of the energy system toward a sustainable system in 2016.^34^ From 2009 to 2013, there were five projects in Germany involving CO_2_ methanation at pilot plant or commercial scale with capacity ranging from 25 kW to 6300 kW.^35^

**Fig. 3:**
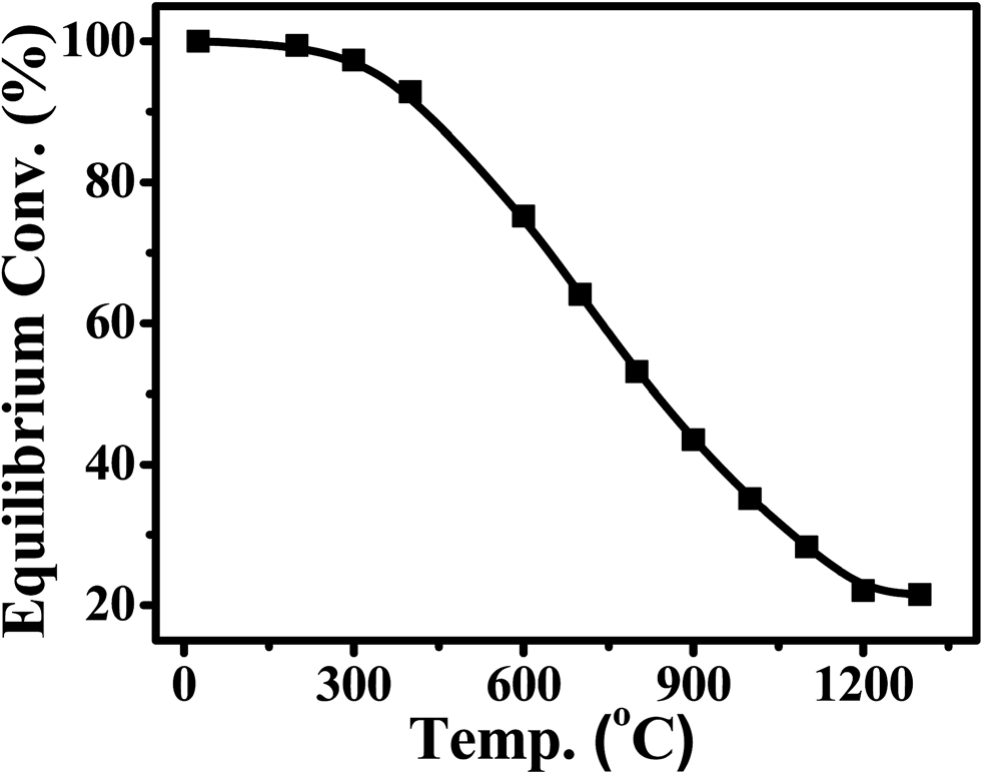
Equilibrium conversion of CO_2_ in methanation at different temperatures (plotted using the data from the literature).^39,40^

CO_2_ methanation was first reported by the French chemist Paul Sabatier in 1902.^36^ Due to the increasing demand for mitigating global warming and storing surplus renewable power, this ancient art has attracted renewed attention. The Sabatier reaction is an advantageous way to store renewable energy such as wind and solar power, to transfer biogas effectively to biomethane, and to convert CO_2_ to chemical feedstocks and fuels.^37,38^ CO_2_ methanation is exothermic with high equilibrium conversion between 25 °C and 400 °C as shown in Fig. 3.^39,40^ CO_2_ methanation can reach 99% CH_4_ selectivity through use of appropriate catalysts, avoid the subsequent product separation and overcome the difficulty of dispersed product distribution. Therefore, such a thermodynamic feature makes CO_2_ methanation more significant in terms of energy efficiency and economic viability.

### 2.1 Metal-based heterogeneous catalysts

CO_2_ methanation can be catalyzed by transition metals such as Co,^41–44^ Ni,^7,45^ Ru,^46,47^ Rh,^48^ and Pd.^49,50^ Based on previously published results, the activity performance of various metal-based catalysts decreases in the following order: Ru > Rh > Ni > Co > Pt > Pd.^34^ Co- and Ni-based catalysts are preferred because of their low cost compared with the noble metals (Ru, Rh, Pd). Ni-based catalysts are the most commonly used for industrial purposes due to their high activity, high CH_4_ selectivity, and easy availability. The catalytic performances of some representative catalysts are summarized in Table 1, as well as the preparation methods and reaction conditions.

**Table 1:** Summary of various catalysts for CO_2_ methanation

Catalyst	Preparation method	Reactor	GHSV [mL h^−1^ g^−1^]	*P* [MPa]	*T* [°C]	*X*_CO_2__ [%]	*S*_CH_4__ [%]	Stability tests	Ref.
NiO–MgO@SiO_2_	Co-precipitation method	Fixed-bed quartz reactor	90 000	0.1	300	80	97	Stable after 100 h	94
NiWMgO_*x*_	Precipitation	Fix-bed quartz tube micro-reactor	40 000	0.1	300	83	99	Stable after 100 h	74
15% Ni 2% La-hydrotalcite	Co-precipitation method	Tubular quartz reactor	12 000	—	250	46.5	99	Stable after 5 h	75
10Ni/Ce–ZrO_2_	Ammonia evaporation method	Fixed-bed quartz reactor	20 000	—	275	55	99.8	Stable after 70 h	88
Ni/CeO_2_	Excess impregnation	Fixed-bed quartz reactor	22 000	0.1	340	91.1	100	Decreased by 18% after 700 min	64
14% Ni 7% Ce/USY	Impregnation	Flow tubular reactor in Pyrex	43 000	0.1	400	68.3	95.1	Stable after 10 h	95
10% Ni/MOF-5	Impregnation	Fixed-bed quartz reactor	2000	0.1	320	75.1	100	Stable after 100 h	84
20% Ni/55% γ-Al_2_O_3_–15% ZrO_2_–15% TiO_2_–15% CeO_2_	Impregnation	Fix-bed quartz reactor	20 000	0.1	300	85	98	Stable after 400 min	79
80% Ni–Al hydrotalcite	Co-precipitation method	Fix-bed quartz reactor	20 000	0.1	300	86	98	Stable after 25 h	80
2.5% Ce–10% Ni/Al_2_O_3_	Ultrasonic impregnation	Fixed-bed reactor	7200	0.1	400	74	98	—	17
12% Ni/Al_2_O_3_ (3DFD structure)	Impregnation, coated on 3DFD structures	Fix-bed quartz tubular reactor	1500	0.1	350	85	98	Stable after 53 h	90
10% Ni/TiO_2_	Dielectric barrier discharge plasma	Fix-bed quartz tubular reactor	60 000	0.1	350	73.2	99	—	91
RQ Ni	Rapid quenching	Capacity hastelloy autoclave	—	3	200	60	99	Five successive cycles without reactivation	78
10Ni3Pr/Al_2_O_3_	Evaporation induced self-assembly	Fix-bed quartz tubular reactor	15 000	0.1	400	76	98	Stable after 50 h	76
0.03% Pt–20% Co–80% Al_2_O_3_	Double flame spray pyrolysis	U-shaped tube reactor	36 000	0.1	400	70	98	—	89
Co/(0.01)PC-600	ZIF-67-derived carbonization	Fix-bed reactor	72 000	3	270	59	99	Stable after 420 min	85
Pt@CSN	Water-soaking-assisted phase-transformation method	Fix-bed reactor	4800	3	320	41.8	95	—	87
10Co/ZrO_2_	Impregnation	Fix-bed reactor	3600	3	400	92.5	99	Stable after 300 h	62
20% Co/KIT-6	Impregnation	Fix-bed quartz tubular reactor	22 000	0.1	260	46	99	—	42
2.5% Ru/P25	Impregnation	Continuous flow fixed-bed reactor	6000	0.1	200	27.4	100	—	67
Ru/CeO_2_	Single-step flame spray pyrolysis	Fix-bed reactor	7640	—	300	83	99	—	66

#### 2.1.1 Metal–support interaction

The traditional catalyst supports are the metal oxides Al_2_O_3_,^21,51^ SiO_2_,^52,53^ ZrO_2_,^54^ TiO_2_,^20,55^ and CeO_2_ (ref. 19) and zeolites.^7^ There are many factors concerning supports that can influence the performance of metal catalysts,^56^ such as pore size,^57^ structure of supports,^42^ surface chemistry, and metal–support interaction.^45,58–60^ Evidently, the activity and selectivity of these supported catalysts are sensitive to the interaction between the active metals and oxide supports.^45,58–60^ Chen *et al.*^61^ in a current perspective provide a bottom-up look at how the synergistic interactions at the metal/oxide interface can tune the reaction mechanisms and in turn the selectivity of CO_2_ hydrogenation. Actually, the metal sites on metal nanoparticles and the M^+^ or O^2−^ sites of oxides are observed to stabilize the key reaction intermediates, *e.g.*, *CO_2_, *C_*x*_H_*y*_, and *C_*x*_H_*y*_O_*z*_ species.

**Fig. 4:**
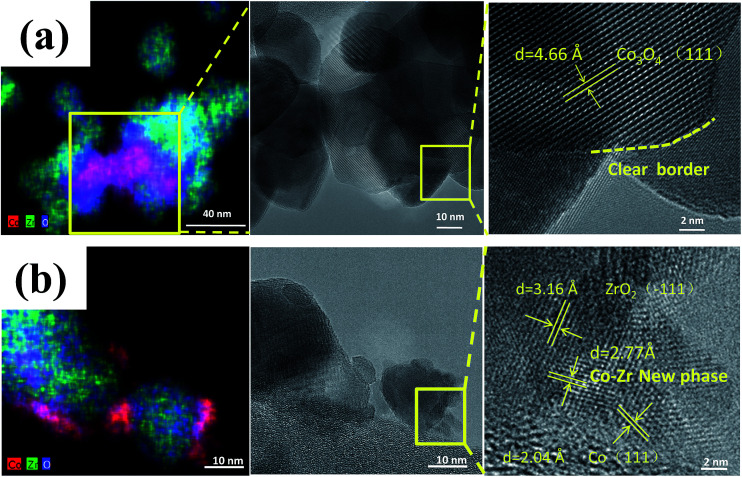
STEM-EDS maps and corresponding TEM images of (a) calcined catalyst precursors Co_3_O_4_/ZrO_2_ and (b) reduced catalyst Co/ZrO_2_. Reprinted with permission from ref. 62. Copyright 2017 Elsevier.

Zhou *et al.*^63^ prepared a series of CeO_2_-supported Ni-based catalysts with various textural properties by hard-template method, soft-template method, and precipitation method, and examined their activity performance in CO_2_ methanation. Among them, they found that the one prepared by the hard-template method exhibited a higher CO_2_ methanation activity, and attributed such superiority to the mesoporous structure and high specific surface area. Furthermore, *in situ* FT-IR and *in situ* XPS results illustrate that the surface oxygen vacancies on the CeO_2_ support were capable of activating the chemisorbed CO_2_ and subsequently forming the CO intermediate.^64^ Martin *et al.*^65^ investigated Rh, Pd, and Ni catalysts supported on different substrates (Al_2_O_3_, CeO_2_, SiO_2_, and zeolites) for CO_2_ methanation. Rh/Al_2_O_3_ and Rh/CeO_2_ exhibited the highest CO_2_ conversion, but differed in mechanism. *In situ* DRIFTS interferograms showed that the linear Rh–CO species was evident on Rh/Al_2_O_3_, suggesting CO_2_ dissociation, while the CO was formed through formate and carbonate intermediate species on Rh/CeO_2_. These advantageous results indicate that the surface oxygen vacancies on the CeO_2_ substrate enabled the interaction with CO_2_, and promoted the CO_2_ hydrogenation. Li *et al.*^62^ prepared Co/ZrO_2_ catalysts for CO_2_ methanation, as well as Co/Al_2_O_3_ catalysts for comparison. The Co/ZrO_2_ catalysts displayed a higher CO_2_ methanation activity with a practically stable performance even after 300 h on stream, while the Co/Al_2_O_3_, in contrast, deactivated rapidly within the same period of time. The Co–Zr interface was observed on the samples in reduced form, which enabled the redistribution of active Co on the ZrO_2_ support due to the special metal–support interaction (Fig. 4). The special Co–Zr interface is crucial for the superior CO_2_ methanation activity. Dreyer *et al.*^66^ have investigated the influence of metal oxide support reducibility on Ru-based catalysts for CO_2_ methanation. They pointed out that the intermediate CO should have an appropriate coverage and strong adsorption, which ensures the occurrence of H_2_ dissociation. The reducible CeO_2_ support is the most suitable to support Ru for CO_2_ methanation compared with the irreducible Al_2_O_3_ which gives a quasi-saturated CO adsorption and limits the co-adsorption of H_2_ and reducible ZnO which has a weak CO adsorption and leads to the reverse water–gas shift (RWGS) reaction.

**Fig. 5:**
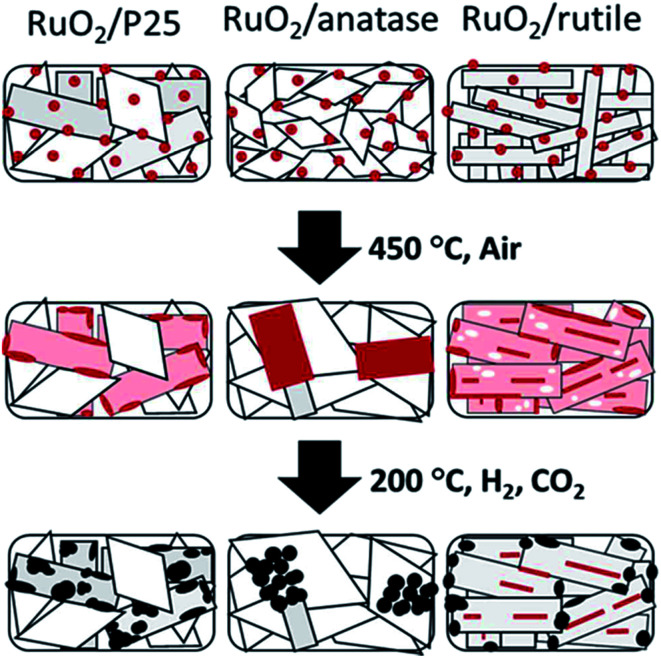
Schematic illustration of the shape evolution of RuO_2_/TiO_2_ catalysts: after RuO_2_ nanoparticle deposition, after thermal annealing at 450 °C, and after reduction and methanation. Red indicates RuO_2_, pink indicates thin RuO_2_ layer, white indicates Ru depleted area, and black indicates metallic Ru. Reprinted with permission from ref. 67. Copyright 2013 RSC.

In addition, metal oxide supports with the same chemical composition and different crystal phase also have an influence on the chemical state of the supported metal. Kim *et al.*^67^ synthesized monodispersed 2 nm RuO_2_ nanoparticle colloidal suspension, and impregnated it onto TiO_2_ with different crystal phases for CO_2_ methanation. The activity and product selectivity were strongly dependent on the composition of different crystal phases of TiO_2_, wherein P25, with 20% of anatase and the rest of rutile, exhibited the highest CO_2_ conversion and CH_4_ selectivity. Inspired by these results, they further developed a fundamental understanding of the composition structure–activity performance relationship.^68^ The phenomenon that RuO_2_ nanoparticles tended to migrate towards rutile TiO_2_ during the CO_2_ methanation process when rutile and anatase TiO_2_ co-existed was evidenced by the stabilization of RuO_2_ on rutile TiO_2_ based on characterization results. Such rutile-favored migration led to the formation of highly dispersed Ru in the reduced form, thereby exhibiting a superior activity (Fig. 5). Lin *et al.*^69^ also observed a similar phenomenon on Ni/TiO_2_ catalysts with different TiO_2_ crystal phases for both CO and CO_2_ methanation. Chen *et al.*^56^ found that PtCo bimetallic catalysts were capable of shifting the selectivity from CO to CH_4_ by altering the oxide supports from TiO_2_ to ZrO_2_, respectively. In other words, PtCo/ZrO_2_ tends to favor CH_4_ formation compared with PtCo/TiO_2_. Both XPS and DFT calculations were carried out to elucidate the origins for CO formation on PtCo/TiO_2_ and CH_4_ formation on PtCo/ZrO_2_. Experimentally, both *HCOO and *HOCO were identified as reaction intermediates on both PtCo/TiO_2_ and PtCo/ZrO_2_, whereas *CH_3_O was only evidenced on PtCo/ZrO_2_. DFT results illustrate that the CO desorption energy is much lower than that of its hydrogenation to *CHO on PtCo/TiO_2_. Therefore, the chemisorbed CO favored desorption energetically rather than the subsequent hydrogenation, thereby leading to a selective production of CO. On the other hand, CO formation was hindered on PtCo/ZrO_2_ catalyst, and CH_4_ was formed. Apparently, the interaction between metal and support plays an important role in product selectivity.

#### 2.1.2 Effect of metal particle size

**Fig. 6:**
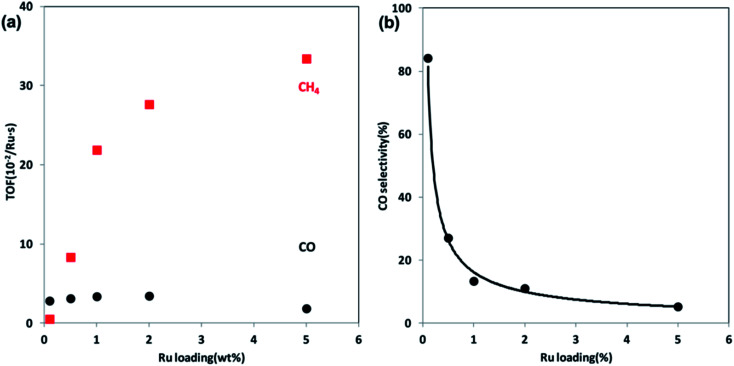
(a) TOFs of CO and CH_4_ formation at steady state at 300 °C over Ru/Al_2_O_3_ catalysts as a function of Ru loading. (b) CO selectivity as a function of Ru loading at 300 °C. Reprinted with permission from ref. 47. Copyright 2013 ACS.

In addition to metal–support interaction, the particle size also strongly affects the kinetic parameters of CO_2_ hydrogenation. Wu *et al.* tested Ni/SiO_2_ catalysts with different metal loadings, namely 0.5 wt% and 10 wt%, in CO_2_ methanation, the loading levels of which corresponded to small Ni clusters and large Ni particles, respectively. CO formation was favored on the small Ni clusters, while more CH_4_ was produced on the large Ni particles.^70^ A similar phenomenon was also evidenced on Ru/Al_2_O_3_ catalysts by Kwak *et al.*,^47^ wherein 1 wt% Ru/Al_2_O_3_ with highly dispersed particles selectively produced CO, and the selectivity was gradually shifted to CH_4_ along with sintering degree of Ru nanoparticles with an increase of Ru loading levels. At 5% of Ru loading, the catalyst had large Ru clusters, therefore making the reaction proceed all the way to CH_4_ (Fig. 6). Iablokov *et al.*^71^ investigated the influence of Co particle size on the activity and selectivity of CO_2_ methanation. A series of near-monodisperse Co nanoparticles with size in the range 3–10 nm were prepared using oleic acid and Co_2_(CO)_8_, among which the larger Co particles exhibited a higher turnover frequency (TOF). Christopher *et al.*^72^ investigated the quantitative relationship between the concentrations of isolated Rh_iso_ active sites and Rh nanoparticles supported on TiO_2_ and the product distribution for CO_2_ thermal reduction. Evidently, the isolated Rh sites favored CO formation, and the CH_4_ selectivity increased with the decrease of Rh_iso_ fraction.

Seen from the results above, both noble and non-noble metal-based catalysts can be applied to CO_2_ methanation, and the experimental results indicate that, within a certain range of metal particle size, the atom-scale structured catalysts tend to favor the RWGS reaction, while the larger metal particles facilitate CH_4_ formation.^50^ To unveil the underlying reasons behind the size-dependent effect, Ma *et al.*^73^ prepared Ir/CeO_2_ catalysts with various Ir loadings using a ligand-free method, and tested them in CO_2_ methanation. The catalysts with low Ir loading presented partially oxidized Ir species, and displayed catalytic selectivity for CO production. On the other hand, more metallic Ir species appeared to emerge when increasing the Ir loading level, leading to a preference for desired CH_4_ formation. Their results suggest that the chemical state of Ir could be finely tuned by altering the loading of the metal. Actually, the particle size effect is the chemical state effect. The metal loading essentially affects the active metal state on the supports and further affects the reaction routes.

#### 2.1.3 Multi-component metal catalysts

The surface properties are also a significant factor in addition to the metal–support interaction and particle size effect. To activate CO_2_ molecules, it is imperative to adjust the surface basicity to improve the adsorption capability towards CO_2_. To achieve this goal, the effect of introducing various rare earth and other transition metals on catalytic properties in CO_2_ methanation has been extensively studied in past decades. Yan *et al.*^74^ demonstrated that W doping can strengthen the Ni–Mg interaction, and enhance NiMgO_*x*_ catalytic performance in terms of CO_2_ methanation activity and stability. Furthermore, such W doping also increased the surface basic sites of NiMgO_*x*_, which could improve CO_2_ stabilization and its subsequent hydrogenation effectively. DRIFTS analysis further demonstrates that these resultant surface basic sites promoted the transfer of adsorbed CO_2_ to monodentate formate species (m-HCOO*) which was proposed as the key intermediate for the CO_2_ methanation.

Similarly, Wierzbicki *et al.*^75^ increased the surface basicity by adding 2 wt% lanthanum to Ni–Mg–Al hydrotalcite-derived catalysts, which remarkably enhanced the CO_2_ methanation activity. In addition, a series of rare earth-doped (La, Ce, Sm, and Pr) Ni-based mesoporous materials were facilely fabricated by the one-pot evaporation-induced self-assembly strategy for low-temperature CO_2_ methanation.^76^ The rare earth-doped catalysts with enhanced surface basicity displayed two or three times higher catalytic activities than the pristine MA-10Ni catalyst in the low-temperature region (200–250 °C). Generally speaking, catalyst basicity improvement increases the CO_2_ adsorption and activation by the second metal addition.

#### 2.1.4 Novel catalysts and process integration

In addition to the traditional metal–support catalysts, more attention has been paid to the incorporation of novel materials with desired features to produce heterogeneous catalysts for CO_2_ hydrogenation, such as multi-metal composite oxides, hydrotalcite, perovskite, and metal–organic framework (MOF)-based catalysts. Depending upon the knowledge and experience collected so far, specific novel materials were intentionally chosen for further modification and/or incorporation with active metals that were already identified as active sites. Preliminary results demonstrate that the tailored materials are capable of improving the catalytic performance as anticipated through strengthened metal–support interaction, generated oxygen vacancies, and improved reducibility of metals. In addition, by DFT calculations, the undercoordinated sites (UCSs) serve as the active centers for hydrogen-assisted CO dissociation and the CO dissociation barrier decreases proportionally with the expansion of the crystal lattice.^77^ Recently, by a rapid quenching technique, Zong *et al.*^78^ fabricated RQ Ni with peculiar UCS-abundant and tensile-strained structural characteristics. This catalyst has superior activity in the low-temperature CO_2_ methanation and the TOF of CO_2_ on RQ Ni is about 8 times that of the highest TOF of CO_2_ ever reported at 473 K. By DFT calculations, the CO activation barrier decreases when the Ni–Ni distance expanded from 2.49 Å to 2.51 Å with tensile strain on the Ni(111) surface. The superior activity conforms to the conclusion that the UCSs are the active centers for CO_*x*_ methanation and more efforts should be aimed at fabricating undercoordinated catalytic materials.

Centi and co-workers have developed γ-Al_2_O_3_–ZrO_2_–TiO_2_–CeO_2_ composite oxide-supported Ni catalysts^79^ and Ni–Al hydrotalcite^80^ catalysts for CO_2_ methanation. A better performance of the catalysts was achieved because of the improvements in the reducibility of active metal Ni.

Metal oxides have relatively low surface areas without featuring pore structures, thereby limiting the intimate contact between reagents and active sites, and even leading to mass transfer limitation. To resolve such issues, some high BET surface supports have been explored. MOFs are a class of crystalline, nanoporous materials that offer such tailorability through large accessible surface areas, tunable pore functionalities, and reactive open metal sites.^81–83^ Zhen *et al.*^84^ prepared Ni-based catalysts using MOF-5 (surface area of 2961 m^2^ g^−1^) as support, and obtained a high Ni dispersion (41.8%). Such 10Ni/MOF-5 catalyst with highly dispersed Ni showed a higher activity than the benchmark Ni/SiO_2_, and presented a superior stability after 100 h on stream for low-temperature CO_2_ methanation. Li *et al.*^85^ prepared ZIF-67-derived Co-based porous carbon catalysts, and achieved both excellent catalytic performance and good stability in comparison to the traditional Al_2_O_3_-supported counterpart. This catalyst even exhibited prominent activity performance in CO_2_ methanation at low temperatures compared with the other Co-based catalysts supported on either metal oxides or zeolites.^41,42,86^ Meanwhile, Zeng *et al.*^87^ have proposed a general synthesis route of ZIF-67-derived bifunctional nanoreactors *via* a water-soaking-assisted phase-transformation method for CO_2_ hydrogenation. They point out that CO_2_ converts to CO on the Pt active sites and CO methanation to CH_4_ occurs on the Co active sites. The Pt@CSN (cobalt silicate nanoparticles) bifunctional nanoreactors increase the CO_2_ conversion and CH_4_ selectivity obviously through prolonging the intermediate retention time on the catalyst surface and enhancing the probability for CO to further convert to CH_4_. These achievements provide insight into adapting these advancements toward the industrial utilization of CO_2_ in terms of economic and sustainable viability. However, MOFs are unfavorable for high-temperature reaction because of their instability under the hydrothermal reaction conditions, especially given that CO_2_ hydrogenation usually requires high temperature. From another point of view, developing low-temperature methanation catalysts with high activity is also a promising way for CO_2_ conversion from the energy conservation perspective.

In addition to the traditional homogeneous catalyst preparation methods, such as impregnation method and co-precipitation method, more and more innovation techniques have been employed to prepare catalytic materials to remedy the defects existing in traditional methods. Kawi and co-workers developed Ni/Ce–ZrO_2_ catalysts by the ammonia evaporation (AE) method, and remarkably improved the Ni reducibility (total H_2_ uptake = 3.37 mmol g^−1^) compared with the impregnation (total H_2_ uptake = 3.32 mmol g^−1^) and deposition–precipitation (total H_2_ uptake = 2.06 mmol g^−1^) methods.^88^ The Ni/Ce–ZrO_2_-AE possessed more oxygen vacancies, and a strengthened metal–support interaction, thereby contributing to the high activity and stability for low-temperature CO_2_ methanation. Schubert *et al.*^89^ used the double flame spray pyrolysis technique to control the Pt content to as low as 0.03 wt%, and improved the catalytic performance of PtCo–Al_2_O_3_ significantly. Protasova *et al.*^90^ manufactured macro-porous catalytic supports by an innovative and highly reproducible robocasting technique, three dimensional fiber deposition (3DFD), and the supports were coated with Ni/Al_2_O_3_ suspension to achieve sufficient catalytic coating. The catalysts coated on 3DFD supports had improved mass and heat transfer properties, and prevented metal sintering efficiently. These advantages were conducive to maintain the stability of the catalysts. Liu *et al.*^91^ attained a better catalytic performance of Ni/TiO_2_ catalysts prepared by dielectric barrier discharge plasma, depending on which more active Ni(111) facets were selectively exposed for CO_2_ methanation.^92^

To accord with practical applications, researchers have recently paid attention to the integration of carbon dioxide capture and utilization processes by incorporating together both CO_2_ capture system and catalytic CO_2_ conversion system. Farrauto^8,93^ and co-workers devoted their effort to exploring dual functional catalysts which enable CO_2_ capture from an emission source, and conversion of it to synthetic natural gas in the same reactor at the same temperature (320 °C). In this process, the catalyst composition comprised 5% Ru 10% CaO/Al_2_O_3_, wherein the components of CaO and Ru were responsible for CO_2_ adsorption and conversion, respectively. Of note, this new approach utilized flue gas sensible heat, and needed no additional heat input, which made it more attractive in mitigating the current energy shortage. Grunwaldt *et al.*^33^ explored Ni-based catalysts under dynamic reaction conditions, especially under a fluctuating supply of renewable H_2_. They found the oxidation of Ni particles occurred after the removal of H_2_ from the gas stream, and a lower catalytic performance was observed consequently. Apparently, the Ni/CaO–Al_2_O_3_ catalyst was unadapted to the dynamic reaction conditions. Such an issue impeded its implementation in real industry, which also made the search for efficient ways for renewable H_2_ supply more important.

### 2.2 Mechanisms of CO_2_ methanation

CO_2_ methanation can be catalyzed through either CO route or formate route, which is determined by the properties of different active metals and supports. The CH_4_ selectivity is likely determined by the competition between the hydrogenation and C–O bond scission reactions of the *H_*x*_CO intermediates. To achieve high CH_4_ selectivity, the binding of *H_*x*_CO species should be strong enough to facilitate C–O bond cleavage.^61^

Duan *et al.*^96^ investigated the effect of oxygen vacancies on the catalytic performance of Rh/CeO_2_ catalysts in CO_2_ methanation, and developed an understanding of its role in the proposed mechanism. The existence of Ce^3+^, surface hydroxyl, and oxygen vacancies on Ru/CeO_2_ was evidenced from operando XANES, IR, and Raman analyses. Steady-state isotope transient kinetic analysis (SSITKA)-type *in situ* DRIFTS was employed to detect the surface intermediates and track their transformation during the reaction process on both Ru/CeO_2_ and Ru/Al_2_O_3_, wherein the latter was introduced as reference, as it barely had any oxygen vacancies. On the Ru/Al_2_O_3_ catalyst, the carbonyl species, originating from the chemisorbed CO*, was observed until 250 °C, and CH_4_ generation occurred within the same temperature range. In contrast, formate and methanol corresponding bands emerged for Ru/CeO_2_, in which the former was identified as a key intermediate *via* this route, and methanol-to-methane transformation was the rate-determining step at a much lower temperature (150 °C). In this work, oxygen vacancies played a crucial role in CO_2_ activation and formate formation. Sharma *et al.*^97^ prepared Ru-substituted Ce_0.95_Ru_0.05_O_2_ catalyst for CO_2_ methanation, and interpreted a plausible reaction mechanism by combining the characterization results (TPR and DRIFTS) and DFT calculation. In this case, surface CO* species, rather than formate, was more likely to act as a key intermediate for CH_4_ production, wherein the reaction proceeded through the following steps: CO_2_ → CO → OCH_2_ → OCH_3_ → CH_4_. Note that this proposed reaction pathway differed from that of the supported metal catalyst (Ru/CeO_2_), which proceeded *via* CO_2_ → CO → HCOO^−^ → C → CH_4_.

**Fig. 7:**
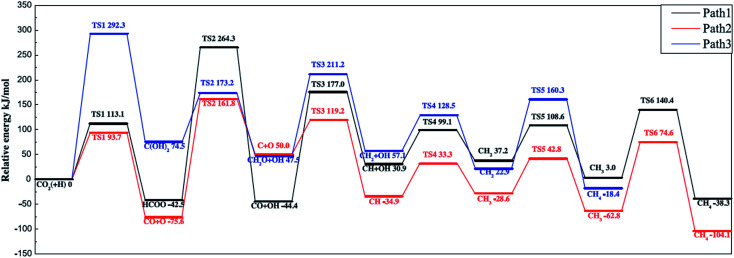
Potential energy diagram of three mechanisms of CO_2_ methanation. Reprinted with permission from ref. 92. Copyright 2015 Elsevier.

**Fig. 8:**
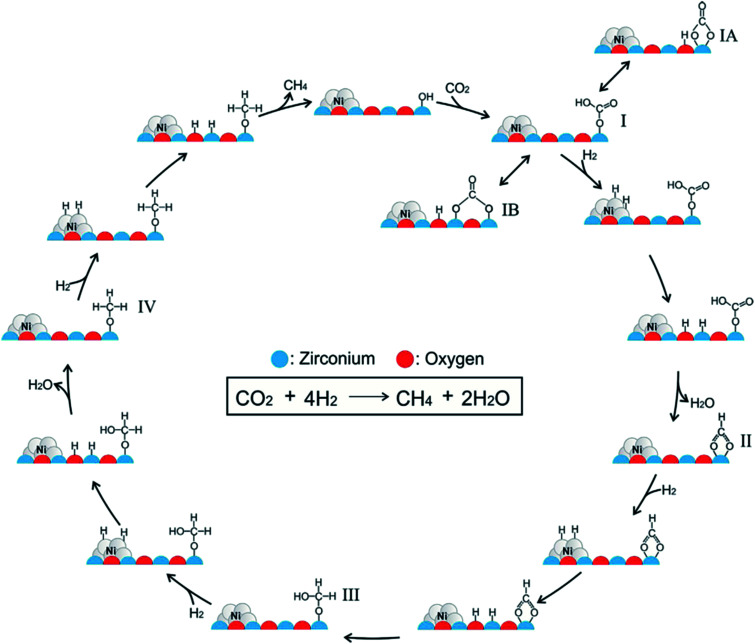
Mechanism of CO_2_ methanation on ZrO_2_-supported Ni catalysts. Reprinted with permission from ref. 99. Copyright 2017 Elsevier.

Ren *et al.*^92^ investigated the mechanisms of CO_2_ methanation on Ni(111) surfaces by DFT through three routes with and without CO formation (Fig. 7). Considering the energy barriers and reaction energies for these different routes, the CO route was more favorable energetically for CO_2_ methanation on Ni(111) surface: CO_2_ → CO + O → C + O + 4H → CH_2_ + 2H → CH_3_ + H → CH_4_. Salmeron *et al.*^98^ also concluded that the methanation reaction proceeded *via* CO intermediate on Ni(111) surface as evidenced by ambient pressure X-ray photoelectron spectroscopy. Meanwhile, they reported that the Ni(110) seemed to convert CO much more easily to atomic carbon than Ni(111). Henriques *et al.*^18^ also investigated the mechanism of USY zeolites-supported Ni catalysts for CO_2_ methanation, and reported results also supporting the CO route. On the other hand, Gonzalez *et al.*^99^ identified the surface species on Ni/ZrO_2_ catalysts and bare ZrO_2_ during CO_2_ methanation using DRIFTS, and they proposed a different scenario with the formate route as the favorable one, as displayed in Fig. 8. In this mechanism, chemisorbed CO_2_ reacted with surface hydroxyl groups of ZrO_2_ to give bicarbonate species that can be reversibly converted to carbonate species. H_2_ was dissociated on the surface of Ni particles, which may migrate to the reducible metal oxide support by a spillover process, resulting in the formation of surface hydroxyl groups, metal–H species, and formate species. Next, the formate species took part in further hydrogenation to form CH_4_.

In contrast to other reports,^18,92^ the Ni/ZrO_2_ catalysts barely presented vibrational bands of carbonyl species on Ni surface. Instead, carbonate and bicarbonate species were identified on both Ni/ZrO_2_ and bare ZrO_2_, and even their subsequent transition to formate species was evidenced. Clearly, the incorporation of Ni and ZrO_2_ was characteristic of bifunctionality for CO_2_ methanation, in which the former metal sites were responsible for providing hydrogen through dissociation, while the latter support accounted for the CO_2_ stabilization and activation. In other words, CO_2_ methanation can be catalyzed through the formate route rather than the CO route on ZrO_2_-supported Ni catalysts.

In sum, the reaction pathway of CO_2_ methanation varied depending on the catalytic system used, and strongly depended on the selection of active metals and supports and their interactions.

### 2.3 Deactivation of CO_2_ methanation catalysts

Deactivation of metal catalysts is a big challenge in CO_2_ methanation. The deactivation of methanation catalysts can be divided into two types: (a) chemical deactivation and (b) physical deactivation.

The chemical deactivation of CO_2_ methanation catalysts is mainly directed toward the decrease of active sites caused by the formation of spinel structure. Li *et al.*^62^ prepared Co/ZrO_2_ catalysts for CO_2_ methanation, as well as Co/Al_2_O_3_ catalysts for comparison. The Co/ZrO_2_ catalysts displayed a higher CO_2_ methanation activity with a practically stable performance even after 300 h on stream, while the Co/Al_2_O_3_, in contrast, deactivated rapidly within the same period of time. The deactivation of the Co/Al_2_O_3_ catalyst was further studied through thermogravimetric analysis and hydrothermal (H_2_O) treatment verification tests. Extra H_2_O was pumped into the reaction system which led to a large amount of CoAl_2_O_4_ being formed and accelerated the Co/Al_2_O_3_ catalyst deactivation. Thus, the product H_2_O promotes the formation of the inactive phase CoAl_2_O_4_, leading to the rapid deactivation of Co/Al_2_O_3_ catalysts. Carbon deposition is one of the reasons for deactivation; however, the main reason for deactivation is the formation of inactive phase CoAl_2_O_4_ spinel structure.

Physical deactivation is caused by carbon deposition and active metal sintering. Kesavana *et al.*^100^ synthesized Ni/YSZ catalysts by different methods. On the Ni/YSZ catalyst obtained by impregnation method, graphitic filaments are formed on Ni^0^ particles exposing flat surfaces, whereas thin layers of carbon are formed on Ni^0^ particles with spherical shape. Ni/YSZ(EDTA) catalyst showed remarkable stability and operando XAS showed that Ni/YSZ(EDTA) catalyst did not undergo deactivation by Ni^0^ → Ni^2+^ oxidation using high CO_2_ : H_2_ ratio. Carbon deposition on the catalyst can be avoided by adding steam or increasing the H_2_/CO_2_ ratio because hydrogen reacts with the carbon deposits and prevents catalyst deactivation. To mitigate metal sintering, common strategies are increasing metal dispersion through strong metal–support interaction,^62^ adding catalyst promoters,^101^ and developing advanced synthesis methods.^89^ Li *et al.*^85^ prepared MOF-derived Co-based porous carbon catalysts in which the active Co particles are separated by the graphite-like carbon avoiding metal sintering effectively. A special catalyst structure can also resist metal sintering effectively. Li *et al.*^94^ prepared NiO–MgO@SiO_2_ core–shell structured catalysts, and activity performance was successfully retained after 100 h on stream. In summary, particles with appropriate size are beneficial for CH_4_ formation.

## 3. CO_2_ hydrogenation to C_2+_ hydrocarbons

CO_2_ hydrogenation to C_2+_ hydrocarbons is of great importance because the long-chain hydrocarbons possess higher energy density, and could be used as fuels and chemicals for a wide range of applications. Utilizing CO as the carbon source for hydrocarbon synthesis through Fischer–Tropsch synthesis has been widespread in industry, and continuing studies to further improve the activity, tune the product distribution, and develop deep understandings of catalytic composition-performance-physicochemical relationships are still ongoing worldwide.^102,103^ Substituting CO with CO_2_ as carbon source makes the reaction thermodynamically more difficult (see the reaction enthalpy below).^104^ Besides, not only will the challenge originate from the chemical inertness of CO_2_, but also from the competition with CO_2_ methanation.^73^ The relatively small amount of CO_2_ adsorbed species compared with dissociated H* on a catalyst surface leads to a low C/H ratio, which favors the fast hydrogenation of surface-adsorbed intermediates and the formation of methane and prevents chain growth.^12,56,105^ Despite the difficulties, the transformation of CO_2_ to value-added chemicals still receives great attention worldwide because of the significance in providing sustainable alternatives to solve urgent issues such as those of energy and the environment. This section will discuss the most recent advances in CO_2_ hydrogenation to hydrocarbons. Similarly, some representative catalysts for CO_2_ hydrogenation to C_2+_ hydrocarbons are presented in Table 2.

**Table 2:** Summary of various catalysts for CO_2_ hydrogenation to C_2+_ hydrocarbons

Catalyst	Preparation method	GHSV [mL h^−1^ g^−1^]	*P* [MPa]	*T* [°C]	*X*_CO_2__ [%]	*S*_CO_ [%]	*S*_CH_4__ [%]	*S*_C_2_–C_4__ [%]	*S*_C_5+__ [%]	O/P	Ref.
Fe_2_O_3_-CT600	Precipitation method	1140	0.15	350	40.0	15^a^	12^a^	37^a^	36^a^	2.7	112
20% Fe/cube-CeO_2_	Incipient wetness impregnation	200	—	390	18.9	73.5^a^	75.5^b^	22.2^b^	1.9^b^	4.1	115
Fe–Co(0.17)/K(1.0)/Al_2_O_3_	Incipient wetness impregnation	3600	1.1	300	31.0	18^a^	13^a^	69^a^		—	116
10K13Fe2Co100ZrO_2_	Electro-spinning method	3600	3	400	42.3	21.9^a^	25.7^a^	34^a^	18.4^a^	4.2	117
CeO_2_–Pt@mSiO_2_–Co	Hydrothermal and impregnation	50 400	0.6	250	2.0	78.0	60.0^b^	40.0^b^	0^b^	—	118
Delafossite-CuFeO_2_	Hydrothermal method	1800	1	300	16.7	31.4^a^	2.4^b^	32.7^b^	64.9^b^	7.7	119
0.05MnFe	One-step sol–gel process	6000	0.1	340	30	7.7^a^	29.3^b^	67.1^b^		0.37	120
ZnGa_2_O_4_/SAPO-34	Physical mixing	5400	2	370	13	46^a^	1^b^	97^b^	2^b^	7.8	121
ZnZrO/SAPO-34	Physical mixing	3600	2	380	12.6	47^a^	3^b^	94^b^	3^b^	5.7	122
In_2_O_3_/HZSM-5	Granule stacking	9000	3	340	13.1	45^a^	1^b^	13.1^b^	78.6^b^	—	26
In–Zr/SAPO-34	Physical mixing	9000	3	400	35	75^a^	5^b^	93^b^	3^b^	6.1	123
Na–Fe_3_O_4_/HZSM-5	Granule mixin	4000	3	320	33.6	14.2^a^	7.9^b^	18.4^b^	73.7^b^	—	105
Fe–Zn–Zr@HZSM-5-Hbeta	Claddingmethod	3000	8	340	14.9	38.6^a^	1.5^a^	71.7^a^	26.8^a^	—	124
Cu–Zn–Al/modified-HB	Physical mixing	1500	0.98	300	27.6	53.4^a^	0.7^a^	43.2^a^	2.3^a^	—	125

a Calculated from equation 
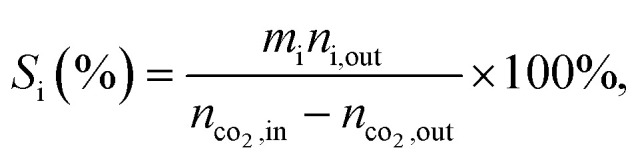
 where *n*_co_2_,in_ and *n*_co_2_,out_ represent the molar concentration of CO_2_ in the feed and effluent, respectively; *n*_i,out_ represents the molar concentration of product i in the effluent; and *m*_i_ represents the number of carbon atoms in product i. Products include CO and hydrocarbons.

b Calculated from equation: 
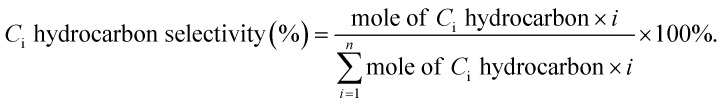

### 3.1 Modified Fischer–Tropsch synthesis (FTS) route

Primarily, CO_2_ hydrogenation to hydrocarbons could proceed through both direct and indirect pathways. The direct way is straightforward conversion of CO_2_ to hydrocarbons^106^ (eqn (1)) or going through the RWGS reaction and FTS reaction (eqn (2) and (3)).^107,108^ In addition to these proposed reaction pathways, some scientists have also attempted to use methanol as a bridge and building unit to synthesize long-chain hydrocarbons *via* CO_2_ hydrogenation, and made a major breakthrough most recently.^96^ This newly developed reaction pathway is another alternative option for CO_2_ hydrogenation to hydrocarbons through an indirect way.

1 CO_2_ HYD, *n*CO_2_ + 3*n*H_2_ = C_*n*_H_2*n*_ + 2*n*H_2_O, Δ_R_*H*_573 K_ = −128 kJ mol^−1^

2 RWGS, CO_2_ + H_2_ = CO + H_2_O, Δ_R_*H*_573 K_ = +38 kJ mol^−1^

3 FTS, 2*n*CO + (3*n* + 2)H_2_ = 2C_*n*_H_2*n*+2_ + 2*n*H_2_O, Δ_R_*H*_573 K_ = −166 kJ mol^−1^

Since converting CO_2_ to hydrocarbons directly makes the reaction kinetically more difficult,^104^ some scientists have turned to the modified FTS route. This section will mainly focus on the major progress made in CO_2_ hydrogenation to C_2+_ hydrocarbons through the modified FTS route. Currently, FTS is mainly catalyzed by Fe-based catalysts because the metal Fe possesses the catalytic characteristic of improving C–C coupling, which was proposed as a rate-determining step.^109^ In 1978, Dwyer *et al.*^110^ found that the presence of CO_2_ impacted the product distribution of FTS on Fe-based catalysts, and such an inspired finding exploited a new field for the development of active catalysts for hydrocarbon synthesis from CO_2_, an alternative carbon source with even greater proportion in the atmosphere. Computational results demonstrate that the kinetics of FTS is not comparable to that of RWGS, which makes it another challenge other than carbon chain growth.^111^ Even so, the similarity between CO and CO_2_ hydrogenation motivated people to apply Fe catalysts, which exhibited good performance in FTS, to CO_2_ hydrogenation at an early stage, and more researchers then endeavored to apply modified Fe catalysts with desired features. Albrecht *et al.*^112^ prepared dopant-free bulk Fe_2_O_3_ by a cellulose-template (CT) synthesis method, and applied it in CO_2_ hydrogenation to hydrocarbons. The catalysts selectively catalyze CO_2_ to C_2_–C_4_ hydrocarbons (selectivity = 37%) with an olefin to paraffin (O/P) ratio of 2.7. Iron carbide, as high as 81 wt%, was detected on the spent Fe_2_O_3_-CT600 catalysts, which was considered as active sites for the FTS.^113,114^ In comparison to FTS, this CT-supported Fe_2_O_3_ catalyst yielded comparable C_2_–C_4_ hydrocarbons, which could be attributed to the improved reducibility and *in situ* formation of iron carbide promoted by the CT-synthesized catalyst precursor.

#### 3.1.1 Selection of support materials

As is well known, the incorporation of a support is able to tune the dispersion of active sites depending on the metal–support interaction. Due to its featured surface chemistry and amphoteric property, Al_2_O_3_ might have a strong interaction with loaded metallic species, and was widely used as support material for the preparation of commercially available catalyst for methanol synthesis.^126^ To increase the Fe dispersion, Al_2_O_3_ was also employed as a support in the preparation of Fe catalysts for CO_2_ hydrogenation to hydrocarbons. Ding *et al.*^127^ prepared a series of FeK/Al_2_O_3_ catalysts, and investigated the effect of surface hydroxyl groups of Al_2_O_3_ on the activity and selectivity of hydrocarbon synthesis *via* CO_2_. Evidently, the variations of both Fe dispersion and particle size were strongly dependent on the point of zero charge (PZC) of the Al_2_O_3_ support. The Fe dispersion increased monotonically with the increase of PZC value, while the particle size showed an opposite trend. The highest CO_2_ conversion (54.4%) and selectivity of C_5+_ hydrocarbons (31.1%) were achieved at PZC = 8.0. ZrO_2_ (ref. 128) and CeO_2_ (ref. 63 and 88) were used as support materials as well due to the basic sites and the oxygen vacancies.^96^ Wang *et al.*^129^ carried out screening tests on catalysts prepared with different types of support materials, including SiO_2_, Al_2_O_3_, TiO_2_, ZrO_2_, mesoporous carbon, and carbon nanotubes (CNTs), among which ZrO_2_ attained the highest selectivity and yield for lower olefins. Chew *et al.*^108^ used N-doped CNTs as a support to prepare Fe-based catalysts (Fe/NCNTs), and O-doped CNT- and SiO_2_-supported catalysts were also employed for comparison. Characterization results demonstrate that the incorporation of NCNTs greatly improved the Fe dispersion and reducibility, which benefited from the improved hydrophilicity and appropriate metal–support interaction. On the other hand, the O-doped SiO_2_ support showed too strong an interaction with the Fe species, which, in turn, exhibited a negative impact on the reducibility of active metal. Murciano *et al.*^115^ prepared Fe catalysts by introducing CeO_2_ with various morphological properties, and examined their influence on the activity and selectivity in CO_2_ hydrogenation to hydrocarbons. Results showed a strong reliance of catalytic performance on the reducibility of Fe species on the support. Among those CeO_2_ materials, the one with cubic morphology helped to improve the reducibility of Fe species as evidenced from the shift of the initial reduction temperature towards lower temperatures, thereby resulting in the obtained highest O/P ratio in comparison to rod-type and nanoparticle-type CeO_2_.

Owing to their tailorable pore structure and featured physicochemical properties, MOFs and their subclass zeolitic imidazolate frameworks (ZIFs) have attracted considerable attention in a diversity of energy-related applications such as CO_2_ capture and even CO_2_ activation.^130–132^ Driven by their unique characteristic, researchers employed this group of materials as supports to prepare heterogeneous catalysts for CO_2_ hydrogenation to hydrocarbons.^84,133^ Some metal-based MOFs and ZIFs, which already were proven to be active for hydrocarbon synthesis from either CO_2_ or CO hydrogenation, were selectively chosen for activity tests in an attempt to collect first-hand data. Guo and coworkers proposed and carried out a series of tests using these novel catalysts, and obtained interesting results. In an early work, they employed MIL-53(Al) and ZIF-8 as supports for preparing α-Fe_2_O_3_ catalysts by a solid grinding method.^133^ Preliminary results indicated that ZIF-8-supported catalysts exhibited a higher CO_2_ conversion than MIL-53-supported ones because the acidity on the MIL-53 impeded CO_2_ adsorption, an important step for heterogeneous catalysis.

#### 3.1.2 Incorporation of promoters in Fe-based catalysts

**Fig. 9:**
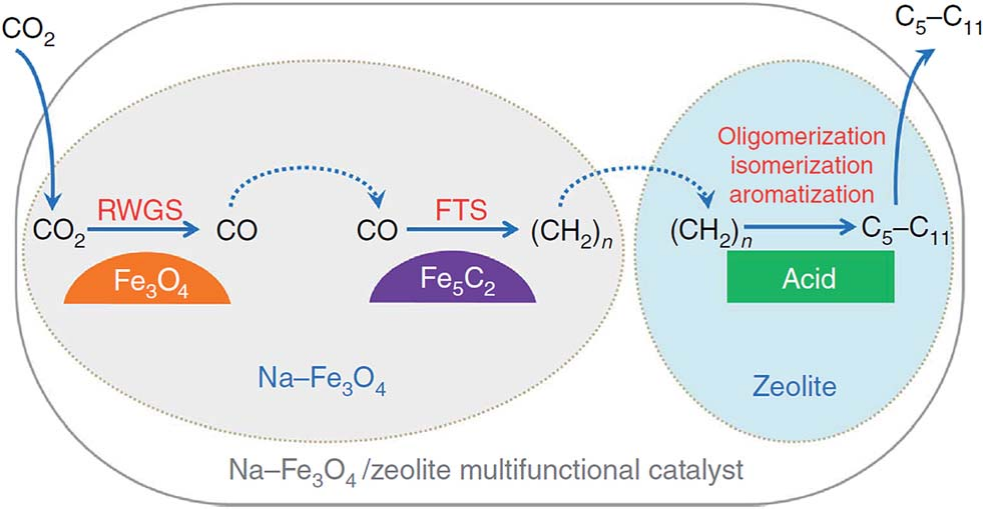
Reaction scheme for CO_2_ hydrogenation to gasoline-range hydrocarbons through modified FTS route. Reprinted with permission from ref. 105. Copyright 2017 Nature.

To further improve the selectivity of higher hydrocarbons, Fe-based catalysts were promoted by a variety of metals, among which K, Na, Cs, Mn, and Cu were representative ones, enabling the enhancement not only of C_2+_ selectivity, but also CO_2_ conversion. Wang *et al.*^129^ added alkali metal ions (with the exception of Li^+^) to Fe/ZrO_2_ catalysts. Among Na^+^, K^+^, and Cs^+^, (0.5–1.0 wt%)K(10 wt%)Fe/ZrO_2_ exhibited the highest CO_2_ conversion. Satthawong *et al.*^116^ investigated the effect of K addition on light olefin production from CO_2_ hydrogenation over Al_2_O_3_-supported Fe–Co bimetallic catalysts. When the K/Fe atomic ratio was 1, the Fe–Co bimetallic catalysts yielded the highest amount of C_2_–C_4_ olefins. Detailed temperature-programed desorption (TPD) analyses demonstrate that the K_2_O on the surface behaved in a manner to suppress the adsorption towards weakly bonded hydrogen which appeared to correlate with the formation of methane. Moreover, the addition of K was able to increase the basicity of the surface, through which more adsorption sites were created on the surface. In other words, the K promoter was capable of shifting the selectivity from methane to desired higher hydrocarbons by tuning the surface H/C ratios. Wei *et al.*^134^ studied the effect of sodium on iron-based catalysts in the CO_2_ hydrogenation process. They reported that sodium can promote the surface basicity of catalysts, which was beneficial for CO_2_ adsorption and the carbonization of Fe_3_O_4_, leading to more C_2_–C_4_ olefins (46.6%) and C_5+_ hydrocarbons (30.1%). Combining the knowledge and experience accumulated, the same group made an exciting breakthrough in CO_2_ conversion to gasoline fuels.^105^ Inspired by the methanol-to-gasoline process and various applications of zeolite in hydrocarbon-related^105^ oligomerization, the Na–Fe_3_O_4_/HZSM-5 catalyst was prepared with the characteristic of selectively producing gasoline components (78%) over methane (4%) of all hydrocarbons at 22% CO_2_ conversion. Characterization results indicate the newly developed catalyst worked in a multifunctional manner, wherein Na, Fe_3_O_4_, Fe_5_C_2_, and acid sites on zeolites were responsible for surface basicity, RWGS, FTS, and oligomerization to hydrocarbons, respectively (Fig. 9). More importantly, instead of functioning separately, granule-mixed catalysts gave a maximum gasoline component selectivity, indicating that selectivity is a function of proximity of the active species. Fierro *et al.*^120^ prepared a series of manganese-iron oxide catalysts with different Mn contents, and found the 0.05MnFe catalyst exhibited the highest CO_2_ conversion and C_2_–C_5_ selectivity. The enhanced activity mainly resulted from the improved reducibility and the Mn-induced promotion of RWGS and FTS reactions. Choi *et al.*^119^ developed a new catalyst prepared by reduction of delafossite-CuFeO_2_ and the catalyst can be transformed *in situ* to active phase χ-Fe_5_C_2_. The CuFeO_2_-derived catalyst selectively generated 65% C_5+_ hydrocarbons with 16.7% CO_2_ conversion, while the reference catalysts derived from bare Fe_2_O_3_, namely CuO–Fe_2_O_3_ mixture and spinel CuFe_2_O_4_, were much less active, and mainly produced light hydrocarbons.

#### 3.1.3 Other metal-based strategies

Fe and Co both display excellent performances in FTS, but Co-based catalysts selectively produce methane when replacing CO with CO_2_ as carbon source.^135^ Lietti *et al.*^136^ developed a deep understanding of the difference between CO and CO_2_ hydrogenation on Co- and Fe-based catalysts. They found that the different adsorption strengths of CO and CO_2_ affected the H/C ratio on different catalyst surfaces, wherein CO_2_ was more active than CO on Co than Fe. If CO_2_ hydrogenation goes through the CO-mediated route, the abundance of chemisorbed CO* is a prerequisite and the Co-based catalysts are the ideal ones.

**Fig. 10:**
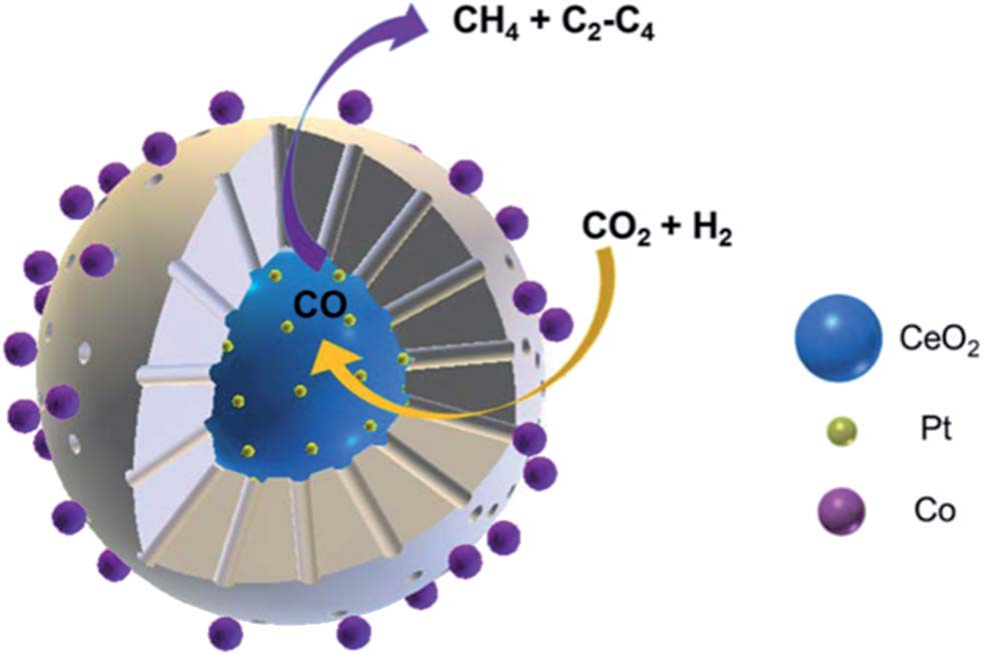
Schematic diagram of CO_2_ hydrogenation on CeO_2_–Pt@mSiO_2_–Co core–shell catalysts. Reprinted with permission from ref. 118. Copyright 2017 ACS.

Yang *et al.*^118^ prepared CeO_2_–Pt@mSiO_2_–Co core–shell catalysts for converting CO_2_ to C_2_–C_4_ hydrocarbons. The two interfaces of Pt–CeO_2_ and Co–SiO_2_ were intentionally created depending on the unique core–shell structure, wherein the former accounted for converting CO_2_ to CO through RWGS, while the latter accounted for the subsequent hydrogenation to C_2_–C_4_ through FTS. Notably, this novel catalyst yielded 60% of C_2_–C_4_ hydrocarbons in total carbon-containing products except CO (Fig. 10).

Another promising strategy to improve the yield of higher hydrocarbons is the application of bimetallic synergy. Satthawong and coworkers^137^ conducted comprehensive screening tests over Fe-M/Al_2_O_3_ (M = Co, Ni, Cu, and Pd) catalysts with fixed M/(M + Fe) atomic ratios at 0.1 at per at and their K-promoted counterparts. The combination of Fe with either Co, Cu, or Pd led to a significant improvement of chain-growth possibility and bimetallic promoting effect on C_2+_ hydrocarbon formation, while the Fe–Ni(0.1) catalysts, on the contrary, selectively produced undesired CH_4_. Interestingly, the combination of Fe and Co, Cu or Pd enhanced the catalyst activity obviously compared with their monometallic counterparts, indicating a strong synergetic effect and intimate proximity existed between the combined metal components. Also, the K addition further increased the CO_2_ conversion and the C_2_–C_4_ olefin production. In following work, the Fe–Co bimetallic catalysts with a wide range of Co/(Fe + Co) atomic ratios (*i.e.*, 0.0–1.0 at per at) were selectively chosen for a systematic examination to unveil the synergetic regime of this bimetallic combination and the function of promoter in terms of adsorption properties.^116,138^ Evidently, doping Fe with appropriate amount of Co (*e.g.*, Co/(Co + Fe) = 0.17 mol mol^−1^) can maximize the promotion of C_2+_ hydrocarbon production. Inspired by such significant bimetallic synergetic effect, Li *et al.*^117^ synthesized Fe–Co–Zr polymetallic fibers for CO_2_ hydrogenation, and obtained 27.5% C_2_–C_4_ olefins with the addition of K.

### 3.2 Methanol-mediated route

In addition to these proposed reaction pathways, some scientists also attempted to use methanol as a bridge and building unit to synthesize long-chain hydrocarbons *via* CO_2_ hydrogenation, and made a major breakthrough most recently.^96^ This newly developed reaction pathway is another alternative option for CO_2_ hydrogenation to hydrocarbons through an indirect way.

**Fig. 11:**
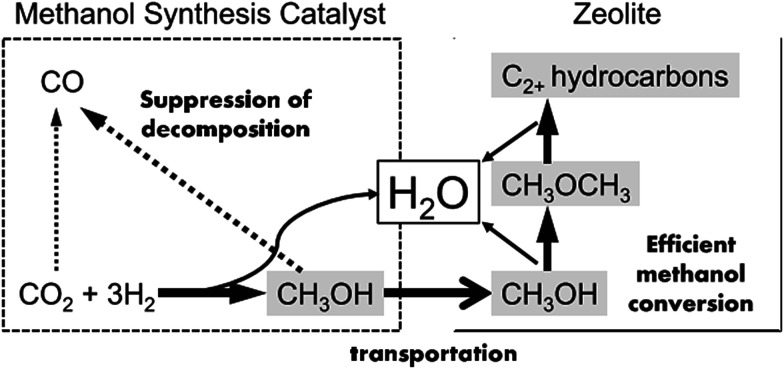
Reaction steps of CO_2_ hydrogenation over composite catalyst (favorable paths are shown in bold lines). Reprinted with permission from ref. 125. Copyright 2015 Elsevier.

The products of FTS generally follow a statistical hydrocarbon distribution, which is known as the Anderson–Schulz–Flory (ASF) distribution.^139^ In the ideal case, the chain-growth probability (*α*) is independent of carbon chain length. Therefore the product distribution is determined by the chain-growth probability (*α*). For example, if CO_2_ hydrogenation is through the modified FTS route, ASF distribution of FTS products limits the maximum selectivities of C_5_–C_11_ (gasoline range) and C_12_–C_20_ (diesel range) hydrocarbons to roughly 45% and 30%, respectively.^140,141^ To break the limitation of ASF distribution and get more gasoline or lower olefins, the methanol-mediated route is an ideal path. Enlightened by the superior selectivity to methanol (*ca.* 100%) on In_2_O_3_-based catalysts from CO_2_ hydrogenation,^142^ Sun *et al.*^26^ used In_2_O_3_/HZSM-5 composite to selectively produce 78.6% C_5_–C_11_ hydrocarbons with a high octane number through the methanol route, and broke the ASF restraint. In the proposed reaction pathway, CO_2_ first was converted to methanol on the In_2_O_3_ surface, which was further transformed to hydrocarbons on HZSM-5 *via* the hydrocarbon-pool mechanism. DFT calculation indicates that the CO_2_ was first chemisorbed on the oxygen vacancies on the In_2_O_3_, and the active site was not the metallic phase. Fujiwara *et al.*^125^ prepared composite catalysts obtained by the physical mixing of Cu–Zn–Al oxide and HB zeolite that was modified with 1,4-bis(hydroxydimethylsilyl)benzene, which were very effective for the production of C_2+_ hydrocarbons, and reached *ca.* 12.6% at 300 °C and 0.98 MPa. They proposed a reaction scheme of CO_2_ hydrogenation over the composite catalyst as illustrated in Fig. 11. The methanol was synthesized on the Cu–Zn–Al oxide catalyst, and methanol was converted to C_2+_ hydrocarbons on the zeolite, which proceeded simultaneously in a single catalytic bed. The preservation of the strong acid sites of the modified HB zeolite with hydrophobic surface improved the second-step CH_3_OH conversion activity.^143^

There are some differences between CO_2_ hydrogenation and CO hydrogenation to hydrocarbons through the methanol-mediated route, such as the molecular polarity,^105^ number of C–O bonds and adsorption capacity of reactants.^144^ However, there are also some similarities, such as the subsequent reaction on the zeolites when methanol is formed. So, the design of bifunctional catalysts for CO_2_ hydrogenation can be inspired by the syngas conversion catalysts. Recently, the products of syngas conversion over the bifunctional catalysts have broken through the traditional ASF distribution and giving the desired products selectively. Wang *et al.*^145,146^ prepared mesoporous H-ZSM-5-supported cobalt nanoparticles for conversion of syngas to hydrocarbons. The C_5_–C_11_ selectivity can reach as high as *ca.* 70% which was due to the hydrocracking/isomerization of the higher hydrocarbons on the Brønsted acidic sites of H-ZSM-5. A series of core–shell catalysts (Fe–Zn–Zr@zeolites) were synthesized by Wang *et al.*^124^ to adjust product distribution, especially in an attempt to improve isoalkane content by the confinement effect. Over 80% isoalkanes among all hydrocarbons were produced on Fe–Zn–Zr@HZSM5-Hbeta catalyst.

Wang and co-workers also integrated the methanol synthesis and methanol-to-olefins reactions with a bifunctional catalyst, Zr–Zn (2 : 1)/SAPO-34. The C_2_–C_4_ olefin selectivity can reach 74% with a CO conversion of 11% at 673 K, thus breaking the limitation of ASF distribution.^103^ Furthermore, Wang *et al.* synthesized ZnGa_2_O_4_/SAPO-34 for CO_2_ hydrogenation to C_2_–C_4_ olefins with a selectivity of 86% using the oxygen vacancies on ZnGa_2_O_4_ to activate CO_2_ molecules.^121^ Additionally, the importance of oxygen vacancies was also evidenced by Sun and co-workers. They prepared a bifunctional catalyst composed of indium–zirconium composite oxide and SAPO-34 zeolite which offered C_2=_–C_4=_ selectivity as high as 80% at more than 35% CO_2_ conversion.^123^ DFT calculations revealed that In_2_O_3_ was a unique catalyst in CO_2_ activation and hydrogenation to methanol with its surface oxygen vacancies and that the reaction followed a mechanism comprising the cyclic creation and annihilation of oxygen vacancies.^147^ These results indicated that the incorporation of Zr into In_2_O_3_ created new kinds of vacancies with high concentration, which progressively enhanced the reaction rate evidenced by DFT calculations. It is worth noting that no obvious deactivation is observed over 150 h, indicating a promising potential for industrial application. Bao *et al.*^148^ presented a process to eliminate the ASF distribution for synthesis gas to light olefins that was enabled by a bifunctional catalyst with two types of active sites. The partially reduced oxide surface (ZnCrO_*x*_) activated CO and H_2_, and C–C coupling was subsequently manipulated within the confined acidic pores of SAPO zeolites. They suggested that the appropriate distance of the two active sites was beneficial for C_2_–C_4_ formation. The C_2=_–C_4=_ selectivity could be optimized as high as 80%, and the C_2_–C_4_ selectivity was 94%, which broke the theoretical limit of only 58% for C_2_–C_4_ hydrocarbons as well. Li *et al.*^122^ also fabricated a tandem catalyst, ZnZrO/SAPO-34, for CO_2_ conversion with a selectivity for lower olefins as high as 80–90% among hydrocarbon products. It is proposed that CO_2_ and H_2_ were activated on ZnZrO and the C–C bond formation was performed on SAPO through DRIFT characterization. CH_*x*_O was considered as intermediate species that included not only methanol. This tandem catalyst showed a resistance to thermal and sulfur treatments (H_2_S and SO_2_), suggesting promising potential application in industry.

The current results have demonstrated that the preparation of bifunctional catalysts combining metal oxides and zeolites is an effective way to control product selectivity for C_1_ conversion, and the appropriate hydrogenation ability of the two components in such bifunctional catalyst is crucial for adjusting product selectivity.

In summary, CO_2_ hydrogenation to C_2+_ hydrocarbons can be catalyzed through modified FTS route or methanol-mediated route to promote hydrocarbon chain growth. For the modified FTS metal-based catalysts, appropriate active metal should be chosen, such as Fe, to get the best hydrogenation capacity. The support basicity and oxygen vacancies should also be improved to increase CO_2_ adsorption and activation.^62^ In addition, adding promoters to adjust the surface C/H ratio and reduction capacity of the active metal is another approach to promote hydrocarbon chain growth.^116,120,135^ For the methanol-mediated route, bifunctional catalysts combining metal oxides and zeolites are crucial for obtaining a higher selectivity of long-chain hydrocarbons. Acid sites are important for the conversion of methanol to hydrocarbons and the channel diameter can influence product selectivities due to the shape-selectivity characteristic.^143^ SPAO-34 with 8-ring pore structure is beneficial for C_2_–C_4_ formation^148^ and ZSM-5 with 10-ring pore structure will lead to C_5_–C_11_ formation.^105^ Therefore, tuning acid strength and pore size plays a significant role in the formation of C_2+_ hydrocarbons and it is a promising direction to promote chain growth.

### 3.3 Mechanisms of C–C coupling

There is an essential and very large difference between CO_2_ methanation and CO_2_ hydrogenation to C_2+_ hydrocarbons, which is the C–C coupling barrier for C_2+_ hydrocarbon formation. In light of the hydrocarbon formation mechanism, the key point for producing long-chain hydrocarbons is controlling the active H/C to an appropriate ratio, wherein too much surface H* will lead to excessive hydrogenation and methane formation, while the opposite condition will offset the hydrogenation ability and reduce the activity of CO_2_ conversion. Satthawong and co-workers added different amounts of potassium to Fe–Co bimetallic catalysts to tune the product selectivity. The CO_2_ adsorption was promoted and the H_2_ adsorption was suppressed with increasing K content through CO_2_-TPD and H_2_-TPD; the C_5+_ selectivity also increased with more potassium addition. However, when the K/Fe atomic ratio increased from 0.5 to 1, the CO_2_ conversion decreased. They attributed the selectivity of the product to the type and concentration of chemisorbed hydrogen and carbon dioxide on the catalyst surface.^116^ Apparently, tuning the surface H/C ratios to manipulate and optimize the product distribution appears to be decisive in the synthesis of C_2+_ hydrocarbons *via* CO_2_ hydrogenation.

The crucial step of hydrocarbon synthesis by CO_2_ hydrogenation is first C–C bond formation and C–O bond cleavage. Many computational studies have been conducted to investigate the C–C coupling step over various catalysts.^149–157^ Different metals showed diverse catalytic performances. Co- and Fe-based catalysts are widely used to catalyze CO_2_ or CO hydrogenation to hydrocarbons.^158–163^ Cu with unfavorable ability for C–O bond cleavage usually converted CO_2_ or CO to C_2+_ hydrocarbons^164^ or alcohols.^28,150,165–168^

#### 3.3.1 C–C coupling over Fe-based catalysts

Fe-based catalysts are widely used in FTS for hydrocarbon production. Due to the similarity, Fe becomes one of the most important components in the catalysts for CO_2_ hydrogenation to hydrocarbons. Pham *et al.*^155,157^ studied CO activation and hydrogenation over χ-Fe_5_C_2_(510) surface, the most exposed one among the various facets due to its stability.^157^ The carbon chain growth was more favorable than CH_4_ formation due to the high barrier of 
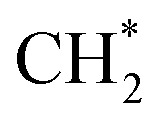
 and 
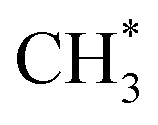
 hydrogenation.^155^ Compared with the CO* insertion mechanism, 
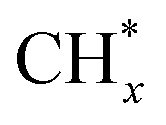
 coupling appeared to be a possible C_2_ hydrocarbon synthesis route on the χ-Fe_5_C_2_(510) surface. The chemisorbed CO* dissociated to become C* on the χ-Fe_5_C_2_(510) surface, and was hydrogenated to CH* species in the following step. C* + CH* and CH* + CH* were the most likely coupling pathways, and were characterized with carbide mechanism.

**Fig. 12:**
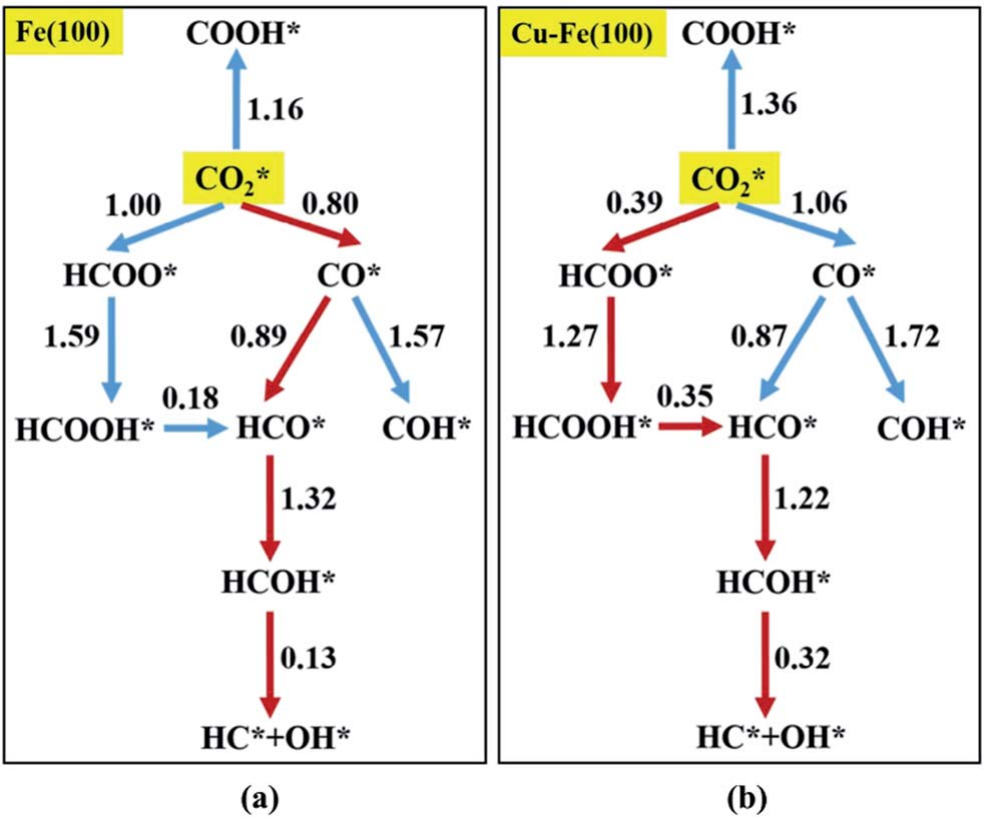
Reaction networks examined to identify energetically favorable C1 species from CO_2_ hydrogenation on (a) Fe(100) and (b) Cu–Fe(100) surface at 4/9 ML Cu coverage. Activation barriers are given in eV (the networks connected with red arrows represent the preferred path for CO_2_ conversion to CH*). Reprinted with permission from ref. 109. Copyright 2017 ACS.

Recently Nie *et al.*^109^ studied C–C coupling over Fe–Cu bimetallic catalysts (Fig. 12). CH* was proposed as the most likely monomer over both pure Fe(100) and Cu-doped Fe(100) surfaces, though CH* formation was quite different over the two surfaces. On the Fe(100) facet, CO_2_ was directly dissociated to form CO* then to CH* through subsequent hydrogenation. Differently, the intermediate CO* was transformed to HCOO* then to CH* through hydrogenation and dissociation in sequence on the Cu-doped Fe(100) facet. The pure Fe(100) favored CH_4_ synthesis with a low barrier of CH* hydrogenation, while Cu promoted C_2_H_4_ synthesis with a low barrier CH* + CH*. The 
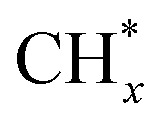
 coupling pathway was also proposed as a plausible mechanism for C_2_ hydrocarbon synthesis over other iron facets.^169,170^ Clearly, the appropriate catalysts for CO_2_ hydrogenation to hydrocarbons do not always go through the CO route (modified FTS route), which offers an alternative to break the restraint of the ASF distribution and the equilibrium conversion of CO_2_ to methanol.

#### 3.3.2 C–C coupling over Cu-based catalysts

**Fig. 13:**
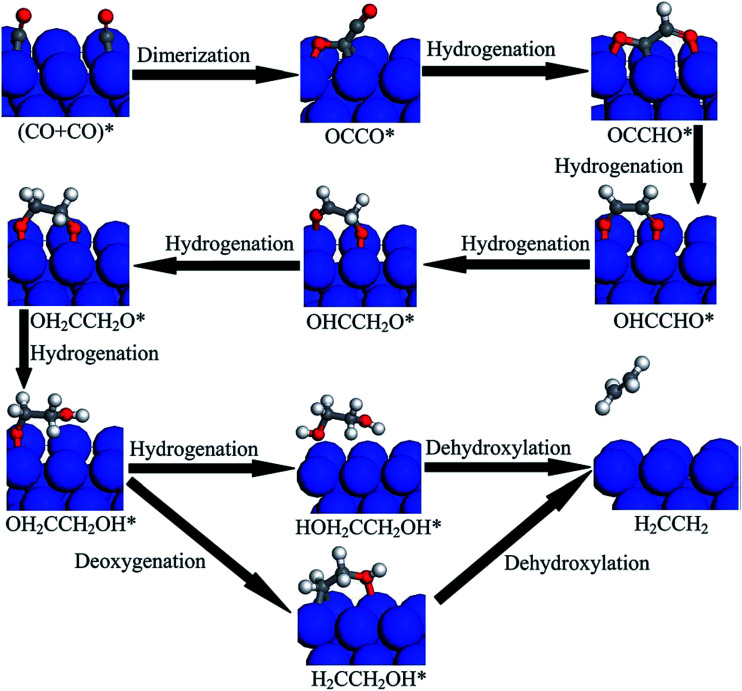
Proposed reduction pathways for the production of C_2_H_4_ in the reduction mechanism of CO dimer on Cu(100). Reprinted with permission from ref. 150. Copyright 2015 RSC.

Cu-based catalysts are widely used for CO_2_ hydrogenation. Ou *et al.*^149,150^ attributed the initial C–C bond formation to CO* dimerization on the Cu(100) facet. The product distribution varied depending on different facets of Cu during the CO_2_ hydrogenation. C_2_H_4_ was formed more favorably on the Cu(100) surface, and CH_4_ was the main product on the Cu(111) surface under chemical conditions. CO was formed through both the direct dissociation of CO_2_ over Cu(100) surface and the dissociative hydrogenation over Cu(111) surface. The CO* dimerization was more favorable than CO* hydrogenation to CHO* in terms of kinetics. The CO* dimer then underwent further hydrogenation to form C_2_H_4_ on the Cu(100) surface as depicted in Fig. 13, while CO* hydrogenation with CHO* as the main intermediate produced CH_4_. Recently Xiao *et al.*^151^ proposed a pH-dependent route for C_1_ and C_2_ product formation over Cu(111) facets. The preferred pathway for C_2_H_4_ formation under aqueous condition was CO → COH → CO–COH → COH–COH → C–COH → C–HCOH → C–CH → C–CH_2_ → CH–CH_2_ → CH–CH_3_ → CH_2_–CH_3_ → CH_2_–CH_2_, which is similar to the CO* dimerization mechanism on the Cu(100) surface.

Compared with the CO* dimerization mechanism, CO* coupled with CH_*x*_ also contributed to hydrocarbon formation over Cu-based catalysts. Wang *et al.*^152–154^ investigated the effect of Cu on higher alcohol and hydrocarbon formation. The higher alcohol formation was facilitated by lowering the barrier of CO* coupling with 
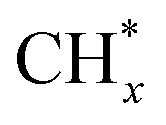
 using copper as the promoter over Co-based catalysts.^153^ The C_2_ oxygenate was the main product over CuΣ5(310) surface by CO* coupling with 
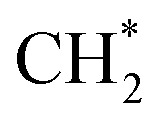
 which could not occur over pure Cu(111) and Cu(100) facets.^152^ The coverage of 
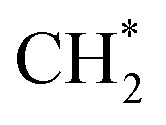
 has an essential role in this mechanism. Cu exhibited much better catalytic performance for the association reaction than Co and Ni, while Co benefited the dissociation reaction.^154,171^ With the assistance of Co and the CuΣ5(310) surface's special active sites, the formation of 
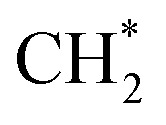
 was accelerated, and the CO* coupling mechanism was favored. Co and Ni were capable of catalyzing CO to CH* and further to 
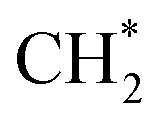
 as the favorable monomer. Due to the low barrier of CO* insertion into 
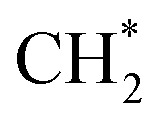
 over Co(111), the Co-based catalysts favored chain growth. On the other hand, Ni-based catalysts were used for CO or CO_2_ methanation with higher barrier of CO* insertion and lower barrier of 
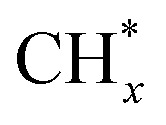
 hydrogenation.^92,154,172,173^ Zhang *et al.*^171^ investigated the CO hydrogenation over Co-decorated Cu alloy catalyst, and stated that the Co–Cu(211) surface was conducive to ethanol formation rather than methane or methanol, and the C–C coupling was accomplished by interacting CO* with 
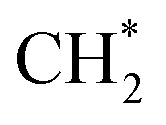
 and 
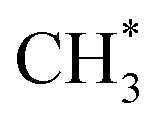
.

Zuo *et al.*^156^ explored ethanol synthesis by syngas over alloy-like CoCu(111) surface, and they found that CO* + 
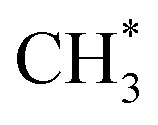
 was the most likely pathway of coupling. The above computational studies demonstrate that CO*, as the main intermediate or the reactant during CO_2_ hydrogenation, was able to interact with surface CO* or 
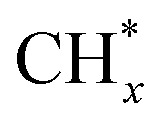
 species over Cu- and Co-based catalysts, through which C–C coupling was available for the formation of long-chain products.

### 3.4 Deactivation of catalysts for CO_2_ hydrogenation to hydrocarbons

Lee *et al.*^174^ investigated the reasons for deactivation of Fe–K/γ-Al_2_O_3_ for CO_2_ hydrogenation to hydrocarbons through XPS, HR-TEM, TPO, and Mössbauer spectroscopy. The reasons for deactivation are different at different positions in the reactor. As time progressed, the Fe_2_O_3_ was reduced to active phase χ-Fe_5_C_3_ and finally the χ-Fe_5_C_3_ was transformed to θ-FeC_3_, which is not an active species for CO_2_ hydrogenation. Hence, in the inlet reactor region, the deactivation pathway was phase transformation. Conversely, the main factor at the outlet part of the reactor was coke deposition.

Li *et al.*^117^ observed the remarkable metal sintering on supported FeCo/ZrO_2_ catalysts which was responsible for the rapid deactivation of activity. In contrast, Fe–Co–Zr polymetallic fibers obtained by a one-step electrospinning technique showed stable activity over the reaction period. Co and Fe were dispersed in proximity to ZrO_2_, but separately from each other, which, in turn, helped reduce the possibility of sintering. Active metals encapsulated in hollow zeolite^175^ or confined in nanotubes^176^ were also applied to resist metal sintering and increase catalyst stability, which are good references for CO_2_ hydrogenation catalysts.

## 4. Conclusion and prospects

Environmental issues have pushed the necessity to reduce CO_2_ emissions caused by the use of fossil fuels. Many efforts have been made to develop catalysts and understand the reaction mechanisms. Heterogeneous thermocatalysis is a promising direction for application in CO_2_ conversion. The catalyst performance can be affected by many factors, such as metal–support interaction, metal particle size and promoters. Ni-based catalysts are mainly used in CH_4_ production from CO_2_ hydrogenation. In addition, Co, Ru, Ir and Rh are also applied for CO_2_ methanation. Fe is an active metal for CO_2_ hydrogenation to C_2+_ hydrocarbons through modified FTS route or methanol-mediated route. Fe–metal bimetallic catalysts have shown markedly improved performance. The preparation of bifunctional catalysts combining metal oxides and zeolites is an effective way to control the product selectivity for C1 conversion. Some experiments and DFT calculations have given the encouraging result that CO_2_ conversion can be catalyzed through the formate intermediate route which is neither the CO route nor the methanol route, which will not be limited by the ASF distribution and the equilibrium conversion of CO_2_ to methanol. The crucial mechanisms of the initial C–C bond formation and C–O bond cleavage are different between Fe-based catalysts and Cu-based catalysts in DFT calculations.

In general, future research directions for CO_2_ hydrogenation are proposed as follows:

1. To adjust the catalyst surface H/C ratio and facilitate C–C coupling and generate high-value-added products.

2. To improve the support basicity and oxygen vacancies and increase the CO_2_ adsorption and activation.

3. To explore more novel catalytic materials and improve the catalyst stability.

4. To explore more active catalysts for low-temperature and energy-saving CO_2_ hydrogenation.

## Conflicts of interest

There are no conflicts to declare.

## Supplementary Material
